# European Network of Breast Development and Cancer turned 10 years: a growing family of mammary gland researchers

**DOI:** 10.1186/s13058-018-1032-9

**Published:** 2018-09-04

**Authors:** Zuzana Koledova, Beatrice A. Howard, Johanna Englund, Karsten Bach, Mohammed Bentires-Alj, Eva Gonzalez-Suarez

**Affiliations:** 1 Department of Histology and Embryology, Faculty of Medicine, Masaryk University, Kamenice 126/3, 625 00 Brno, Czech Republic; 2 The Breast Cancer Now Toby Robins Research Centre, The Institute of Cancer Research, London, UK; 3 Institute of Biotechnology and HiLIFE, University of Helsinki, Helsinki, Finland; 4 Department of Pharmacology, University of Cambridge, Cambridge, CB2 1PD UK; 5 Cancer Research UK Cambridge Cancer Centre, Cambridge, CB2 0RE UK; 6 Department of Biomedicine, University of Basel, University Hospital Basel, Hebelstrasse 20, CH-4031 Basel, Switzerland; 7 Cancer Epigenetics and Biology Program (PEBC), Bellvitge Biomedical Research Institute (IDIBELL), Avinguda de la Gran Via, 199 – 203, L’Hospitalet deLlobregat, 08908 Barcelona, Spain

**Keywords:** Tumor immunology, Myeloid cells and neutrophils, Single cell RNAseq, Imaging, European Network of Breast Development and Cancer Laboratories

## Abstract

The European Network for Breast Development and Cancer (ENBDC), a worldwide network (http://www.enbdc.org/), celebrated its tenth anniversary with a fantastic meeting last March 15–17, 2018 in Weggis with 76 attendees.

During the past 10 years European scientists working on breast development and cancer have met yearly in Weggis to share their knowledge on novel technologies and procedures as well as the most recent scientific achievements in the field. The European Network of Breast Development and Cancer (ENBDC) community has been growing steadily, with members from 13 different European countries, as well as non-European members and attendees of the ENBDC yearly workshop.

The chair of this year’s meeting, Eva Gonzalez Suarez (PEBC, IDIBELL, Barcelona, Spain), together with the PhD/postdoc chairs, Karsten Bach (University of Cambridge, UK) and Johanna Englund (University of Helsinki, Finland) have put together an amazing program. The meeting was organized in four lecture sessions with a total of nine speakers, opening and closing keynote sessions, and six short talks selected from the abstracts. Two poster sessions, with a total of 47 posters, allowed an active role for every attendee.

In this tenth anniversary one of our goals was to encourage young scientists to pursue their careers in science and maintain research excellence in mammary gland biology and breast cancer in Europe and worldwide. Towards this aim we invited as a keynote speaker Zena Werb (University of California San Francisco, USA), whose revolutionary work in the mammary gland and breast cancer field has been inspirational for many scientists worldwide. Zena Werb and the rest of the speakers have contributed to mentoring, motivating, and inspiring the next generation of scientists.

Zena Werb discussed her recent findings using single cell RNAseq analyses of human mammary epithelial cells [1]. This technology allows the reconstitution of differentiation trajectories, clarifying the developmental hierarchy of mammary subpopulations. Her data reveal the existence of multiple basal and luminal human mammary subpopulations, highlighting a heterogeneity that is starting to be appreciated. These results nicely complement similar studies performed on the mouse mammary gland [2, 3]. Single cell gene expression analyses in metastatic cells from tissues with low and high metastatic burden revealed how early stage metastatic cells possess a distinct stem-like gene expression signature. By contrast, metastatic cells from high burden metastatic sites were similar to primary tumors [4]. Phylogenic lineage analyses and pseudotime ordering revealed that low burden metastatic cells exit dormancy and enter the cell cycle upon upregulation of c-MYC. These c-MYC-positive cells can be killed with the CDK inhibitor dinaciclib. She finished her presentation by describing cancer as a systemic disease, as revealed by the expansion of neutrophils in peripheral tissues prior to metastasis detection, and showing that metastasis can be reduced by blockage of G-CSF [5, 6].

The first session, on Biomechanics and Imaging, brought insights into the fundamental mechanisms of cell mechanosensing and mammary gland development. Mechanical properties of extracellular matrix (ECM) regulate normal mammary gland development and pathological processes, including breast cancer progression [7]. Therefore, it is of high interest to understand how the cells detect, transmit, and respond to mechanical signals (mechanosensing and mechanosignaling). Pere Roca-Cusachs (IBEC, Barcelona, Spain) introduced the model of a dynamic molecular clutch of actin–talin–integrin–fibronectin, which explains how matrix rigidity and density results in force transmission and transduction. His team identified talin unfolding as the critical mechanism that triggers force transduction. Above a stiffness threshold talin unfolds and binds to vinculin, leading to adhesion growth and nuclear translocation of YAP [8], a mechanosensitive transcriptional regulator with critical roles in development, regeneration, and cancer. Furthermore, Roca-Cusachs’ team demonstrated that YAP nuclear translocation can be driven directly by force applied to the nucleus [9]. Force transmission from the cytoskeleton to the nucleus leads to nuclear flattening, which reduces mechanical restriction in nuclear pores and increases YAP nuclear import. Importantly, their findings revealed a novel mechanosensing mechanism directly converting force into nuclear molecular import that may play a central role in transcriptional regulation in response to mechanical cues. Future work should resolve how mechanosensing is regulated in complex, three-dimensional (3D) multi-cellular environments such as the mammary gland.

Colinda Scheele from Jacco van Rheenen laboratory (Netherlands Cancer Institute, Amsterdam, Netherlands) caught the attention of the ENBDC attendees with impressive intravital two-photon microscopy videos and images of mouse mammary glands and talked about the mechanism of mammary branching morphogenesis during puberty. They and their collaborators from the laboratory of Benjamin D. Simons (University of Cambridge, UK) used whole-mount analysis of mammary epithelial branching patterns, large-scale mammary gland reconstructions, proliferation kinetics measurements, and mathematical modeling to clarify the rules of mammary branched network topology and spatial patterning formation. Their results showed that all mammary trees follow three universal branching rules: ductal elongation only takes places in the terminal end bud (TEB), bifurcation only happens in the TEB, and a branch is terminated if it comes closer to another branch [10, 11]. Moreover, they found that these rules (that ductal tips grow and branch as a default state and stop dividing in high-density regions) also apply to pattern formation in other branched organs, including kidney and prostate [10, 11]. Intriguingly, despite evidence on side branching from other studies, they did not observe any side branching during pubertal mammary gland development in their intravital imaging work, nor did their mathematical modeling provide support for such a mechanism. Future work needs to clarify this discrepancy.

The second session, on Mechanisms of Mammary Tumorigenesis, started with a talk by Marie Ange Deugnier (Institut Curie, Paris, France) in which she presented their findings on the role of podoplanin (*Pdpn*) in mammary gland development and tumorigenesis. They identified PDPN as a specific marker of basal cells, including multipotent stem cells, that positively regulates the Wnt/β-catenin signaling pathway [12]. P-ERM, RhoGTPases, and changes in the cytoskeleton actin fibers could mediate these effects. Moreover, loss of *Pdpn* attenuated tumorigenesis in a mouse model of β-catenin-induced breast cancer by limiting tumor-initiating cell expansion. Taken together, their work defines *Pdpn* as a regulator of mammary stem cell function and tumorigenesis by potentiating Wnt/β-catenin signaling [12]. Future work should evaluate the clinical importance of *Pdpn* and its possible use as a marker for progression from in situ to invasive breast cancer.

Tumor recurrence is the leading cause of breast cancer-related death. Recurrences arise from residual cancer cells which survive initial therapeutic intervention. The characteristics of these cells, also referred to as minimal residual disease (MRD), have been elusive due to difficulties in obtaining clinical samples of MRD. To shed some light on MRD, the group of Martin Jechlinger (EMBL, Heidelberg and Monterotondo) employed mouse models and organoid culture models to mimic residual disease and recurrence. In these models tissue-specific induction of oncogene expression (*Myc*/*Neu* or *My*c/*Kras*^*G12D*^) results in invasive carcinomas (or their 3D culture equivalent, large acini with filled lumen). Following oncogene inactivation, the tumors completely regress to a non-palpable state (polarized acini in 3D culture) and, with time, spontaneous tumor recurrences develop. Using these models Martin Jechlinger’s group discovered that residual cells acquire a transcriptionally distinct state from normal epithelium and primary tumors, which results in altered lipid metabolism and elevated reactive oxygen species (ROS) [13]. Subsequently, increased oxidative DNA damage potentiates the acquisition of somatic mutations during hormonal-induced expansion of the mammary cell population and leads to tumor recurrence [13]. Importantly, these newly discovered characteristics were also detected in residual cancer cells from neoadjuvant-treated breast cancer patients and provide an opportunity for therapeutic intervention. As Martin Jechlinger showed, reduction of ROS levels and proliferation of mammary epithelium attenuated recurrence in vitro and in vivo [13].

Jeff Pollard (MRC Centre for Reproductive Health, Edinburgh, UK) opened the Tumor Immunology session discussing the effect of the tumor microenvironment in tumor progression. Although it has been established that the tumor microenvironment can suppress tumor phenotypes, a large body of work produced from Pollard’s laboratory and many others have clearly demonstrated that macrophages can promote tumor progression and metastasis [14]. Studies into the mechanisms behind pro-tumoral effects of macrophages on metastatic disease in breast cancer show that macrophages promote tumor cell extravasation, survival, and persistent growth in the lung. Several macrophage-tumor cell-signaling pathways that result in the enhancement of metastasis were highlighted, including the ligands CCL2, VEGF, and CSF-1 and their respective receptors [15]. Recruited macrophages were examined via imaging windows in the lung that allow visualization of extravasation using multiphoton imaging [16]. Cancer cells modify their protrusion activity prior to extravasation and appear to attach to collagen fibers during the process of egress from the lung capillaries. Tracking the fate of recruited classic monocytes, novel immune suppressive precursors of metastasis-associated macrophages were described in the tissue [17]. These immunosuppressive cells suppress the cytotoxicity of activated CD8+ cells partially through superoxide production and can be targeted to prevent breast cancer metastasis. Signals derived from metastasis-associated macrophages determine the metastatic ability of cancer cells. In a recent work from Pollard’s laboratory he has also shown the fate of recruited monocytes in primary tumors which uni-directionally transit through to perivascular macrophages that promote tumor cell intravasation [5]. Knowledge of these mechanisms of monocyte recruitment, retention, and differentiation to mature macrophages that promote tumor progression could lead to therapeutic opportunities to inhibit metastasis that kills most cancer patients.

Cellular surveillance (apoptosis, senescence) and immune surveillance (cytotoxicity) protect us from cancer. Tumor cells feature a plethora of different mechanisms to escape immune surveillance, including loss of MHC1 expression, secretion of the immunosuppressive cytokine TGFβ, and recruitment of immunosuppressive cells: myeloid derived suppressor cells and T regulatory cells (Tregs) [18]. Christophe Caux (Centre Leon Berard, Lyon, France) spoke about mechanisms of Treg-mediated suppression in breast tumors and targeting of the tumor and its immune environment [19]. Caux reported that Treg infiltrating (Ti-Treg) breast cancers have a negative impact on patient outcome. Ti-Tregs are selectively recruited into the tumor environment through CCL22 secretion and expand after ICOS engagement by dendritic cells. Ti-Tregs express high levels of CD39, an ectonucleotidase that cooperates with CD73 to degrade ATP into immunosuppressive adenosine (Ado), which inhibits most inflammatory cytokines except IL-17A. CD73 defines a subset of effector CD4+ T cells (Teffs). CD39+ Tregs selectively target CD73+ Teffs through cooperative degradation of ATP into Ado, which inhibits and restricts the ability of CD73+ Teffs to secrete IL-17A. CD73+ Teffs that infiltrate breast tumors are inactivated by Tregs expressing high levels of CD39 and ATPase activity. Tumor-infiltrating CD73+ Teffs fail to express inhibitory immune checkpoints, suggesting that CD73 might be selected under pressure from immune checkpoint blockade therapy and could represent a target for restoring anti-tumor immunity based on the reactivation of immune surveillance [19].

Emerging evidence indicates that neutrophils’ diverse phenotypic and functional polarization states are able to alter tumor behavior. Cancer patients with high neutrophil to lymphocyte ratios in circulation are at increased risk of developing recurrence disease years later [20] . The work of Karin de Visser (Netherlands Cancer Institute, Amsterdam, Netherlands) demonstrated that tumors induce systemic neutrophil expansion and polarization by stimulating a CCL2/IL-1β/IL-17/G-CSF cascade that involves macrophages and gamma-delta T cells [21, 22]. Neutrophil depletion drastically impairs lymph node and lung metastasis without impacting primary tumor growth. Next, Karin presented a comprehensive study of 16 genetically engineered mouse (GEM) tumor models developed by her collaborator, Jos Jonkers (NKI, Amsterdam), in which hallmarks of systemic inflammation during tumorigenesis were assessed. The p53 status of mammary tumors strongly correlates with neutrophil abundance. Mice with p53-deficient tumors exhibit extensive neutrophil expansion while those with tumors with functional p53 show only moderate neutrophil expansion. Neutrophil accumulation in mice bearing p53-deficient mammary tumors was associated with elevated IL-1β, IL-17, G-CSF, and CCL2 levels in serum. Macrophages grown in conditioned medium from tumor cells with p53 deletions are induced to secrete IL-1β, a potent inflammatory cytokine, suggesting macrophages are altered by the p53 mutant secretome. Analysis of the unique panel of GEM models using RNAseq data leads to clustering based on their p53 status. A number of secreted factors are up-regulated in p53-deficient tumors and cell lines and studies are ongoing to dissect which of these mediators may drive the pro-metastatic systemic neutrophilic inflammation in hosts bearing p53-deficient mammary tumors. The IL-1β/neutrophil axis represents an exciting potential target to inhibit pro-metastatic systemic inflammation.

The last session of the meeting was focused on the effect of the microenvironment in regulation of morphogenesis and tumorigenesis in the mammary gland. Charles Streuli (University of Manchester, UK) described his work on how the cellular microenvironment regulates the circadian clock in mammary epithelial cells. Data obtained in collaboration with Qing-Jun Meng revealed several mammary epithelial genes that exhibit oscillating (circadian) expression. Furthermore, he showed that the circadian clock is required for mammary stem cell function, which is compromised during aging due to the stroma surrounding ducts becoming stiffer [23]. Interestingly, while a stiff environment dampens rhythmic gene expression in the mammary epithelia, the situation is the opposite in fibroblasts, where oscillations are stronger in stiffer matrices [24]. Thus, epithelial and stromal circadian clocks are inversely regulated by their mechano-matrix environment.

Providing evidence for the importance of immunotherapy in cancer, the talk of Xiang Zhang (Baylor College of Medicine, Texas, USA) described his studies on the tumor immune microenvironment. His group recently demonstrated that Th1 CD4+ T cells have a crucial effect in vessel normalization during tumor progression. While disruption of vessel normalization reduced T-lymphocyte infiltration, reciprocal deletion or inactivation of infiltrating CD4+ T cells decreased vessel normalization, indicating a mutually regulatory loop [25]. Despite the enormous success of immunotherapy in some tumors, the individual patient response is highly variable due to the tremendous inter-patient heterogeneity and the immunological hotness or coldness of the tumors. Heterogeneity of the tumor microenvironment is comparable to that of the cancer cells themselves. It is now clear that myeloid cells, comprising tumor-associated macrophages, neutrophils, and myeloid-derived suppressor cells can have pleiotropic effects in tumorigenesis and progression [26]. Using large-scale datasets such as The Cancer Genome Atlas, he showed that not only the composition but also phenotypes of the myeloid cells in the tumor microenvironment vary substantially between patients and can predict outcome, with a large proportion of breast tumors being included in the “cold” cluster. Breast cancer can be subtyped in a bimodal distribution based on the predominant myeloid population that infiltrates the tumor, mainly tumor infiltrating neutrophils and macrophages. This composition is related to metastatic ability, as well as response to treatments, including immunotherapy (Kim et al., unpublished).

The meeting in Weggis came to an end with the keynote talk by its founding father, Mohamed Bentires-Alj (University of Basel, Switzerland). His talk began with a review on the tremendous success of the past 10 years for ENBDC. The meeting that started as a small meeting focusing on methods has progressed to a worldwide community of mammary gland biologists and breast cancer research. He then discussed the challenges and opportunities of targeted therapy in the light of tumor heterogeneity. First, he provided evidence that targeting SHP2 could have therapeutic potential for HER2-positive and triple-negative breast cancer patients [27, 28]. SHP2 is required for the activation of the ERK/MAPK signaling pathway and controls the expression of stemness-related genes. His data show that SHP2 is important for self-renewal of breast tumor-initiating cells and for tumor maintenance and progression. These studies provide new insights into signaling cascades that regulate neoplastic breast stem cells and a rationale for targeting SHP2 in breast cancer. He used this example to stress the importance of the model systems that are used, as most of the phenotypes can only be seen in 3D culture and not in conventional 2D cultures. Finally, he presented published data on the metastasis-promoting activity of the chemokine CCL2 [29]. His studies call for caution when using anti-CCL2 monotherapy as its discontinuation can increase metastasis formation in an IL-6-dependent manner.

## Conclusions

The tenth anniversary ENBDC meeting was, once again, a great success. All attendees have benefitted from the small number of participants, the methods-based talks, and the opportunity to discuss the field with experts. The informal talks and familiar atmosphere fostered the interaction and encouraged everyone to discuss their unpublished work. Despite breast cancer being considered a poorly immunogenic tumor type it cannot escape the tremendous revolution of tumor immunotherapy. This was the main topic of this year’s meeting, with several talks highlighting the relevance of myeloid cells, macrophages, and neutrophils. The DeOme award for the best short talk went to Sara Pensa (University of Cambridge, UK). The 11th ENBDC meeting is set for May 16–18, 2019 and will be chaired by Zuzana Koledova (Masaryk University in Brno, Czech Republic).
